# Trained immunity-based mucosal immunotherapies for preventing respiratory infections: a translational, evidence-graded appraisal of platforms, populations, and the protection–immunopathology balance

**DOI:** 10.3389/fmed.2026.1950422

**Published:** 2026-09-24

**Authors:** Yanhong LYU, Fengjun Zhang, Qiong Zhao

**Affiliations:** 1 Department of Health Management Center, Qilu Hospital of Shandong University, Jinan, Shandong, China; 2 Department of Pulmonary Diseases, Shuguang Hospital Affiliated to Shanghai University of Traditional Chinese Medicine, Shanghai, China; 3 Department of Traditional Chinese Medicine, Shandong Provincial Hospital Affiliated to Shandong First Medical University, Jinan, Shandong, China

**Keywords:** BCG, disease tolerance, mucosal immunotherapy, MV130, respiratory infections, trained immunity

## Abstract

Conventional respiratory vaccines protect through antigen-specific immunity, leaving broad gaps against antigenically diverse and emerging pathogens and inducing little protection at the airway surface itself. Trained immunity, the durable epigenetic and metabolic reprogramming of innate immune cells and their bone marrow progenitors, offers antigen-agnostic, heterologous protection and can be induced directly at the respiratory mucosa, providing a mechanistic rationale for a new class of prophylactic immunotherapies. Here we appraise this field from a clinical-translational, evidence-graded standpoint, integrating three interdependent axes. We first compare the principal inducer platforms (bacille Calmette-Guérin, *β*-glucan and other pattern-recognition-receptor ligands, inactivated polybacterial preparations, and viral vectors) together with the intranasal, sublingual, and inhaled routes through which they deposit innate memory, showing that platform and route jointly govern the quality, location, and durability of protection. We then grade the clinical evidence population by population, which forms a gradient rather than a uniform endorsement: strongest for sublingual MV130 in childhood recurrent wheeze, moderate for bacille Calmette-Guérin in older adults and for MV130 in chronic obstructive pulmonary disease, and preliminary but promising in immunocompromised hosts. Throughout, we apply the protection-vs.-immunopathology balance as a critical lens, distinguishing host resistance from disease tolerance and highlighting the biomarkers and patient-selection strategies needed to steer benefit over harm. We conclude that trained immunity-based mucosal immunotherapy is best advanced not as a uniform promise but as a disciplined program aligning standardized interventions, mechanism-matched trial endpoints, and prospective biomarkers, positioning it as a credible complement to antigen-specific respiratory vaccines.

## 1 Introduction

Respiratory infections remain among the heaviest infectious burdens on human health, causing millions of deaths each year and driving a large share of global antibiotic use (1). Licensed vaccines have sharply reduced severe disease for several respiratory pathogens, yet their protection is anchored to cognate antigens. This design leaves recurrent gaps: coverage narrows against antigenically variable or newly emerging pathogens, injectable formulations induce limited immunity at the airway surface, and no single antigen-specific product spans the diverse viral and bacterial causes of respiratory illness (2). A complementary strategy that protects broadly, and at the site of entry, would address a need that conventional vaccinology has not met.

Trained immunity offers a mechanistic route to such breadth. Innate immune cells, long assumed to lack memory, can undergo functional reprogramming after an initial stimulus and respond more vigorously to unrelated secondary challenges (3). This adaptation is driven by epigenetic remodeling and metabolic rewiring of myeloid cells and their bone marrow progenitors, rather than by antigen-specific clonal expansion (4). Because the resulting protection is heterologous, it is well suited to settings where the offending pathogen is unknown, mutates rapidly, or acts in co-infection (5). The prototypical inducers, bacille Calmette-Guérin (BCG) and fungal *β*-glucan, have anchored the concept and motivated its translation into vaccines designed specifically to exploit innate memory.

The airway mucosa is a logical place to apply this idea. Local induction of trained immunity reprograms alveolar macrophages and other airway-resident cells, establishing a state of heightened readiness precisely where respiratory pathogens first arrive (6). Mucosal delivery matters: intranasal administration in pre-clinical rodent models of the inactivated polybacterial preparation MV130 confers lymphocyte-independent, long-lasting protection against experimental viral respiratory infection (7), and inhaled adenoviral-vectored vaccines reprogram human alveolar macrophages in a way that intramuscular delivery does not (8). Route and platform therefore shape not only the magnitude of the trained response but where it is positioned.

Clinical evidence is accumulating, though it is uneven across populations and platforms. Sublingual MV130 reduced wheezing attacks in young children in a phase 3 randomized trial (9), and BCG vaccination lowered the incidence of respiratory infections in older adults (10). These gains are not free of risk. Trained immunity is a double-edged process: the same reprogramming that improves host defense can, if excessive or misdirected, drive immunopathology, and some protective effects reflect enhanced disease tolerance rather than faster pathogen clearance (11). Conflicting BCG results against COVID-19 illustrate how context, dose, and host state determine whether the net effect is beneficial (12). Any clinical case for these interventions must therefore weigh protection against harm, and identify who is likely to benefit.

A recent opinion article argued persuasively that trained immunity-based mucosal immunotherapies deserve consideration as next-generation vaccines, illustrating the case mainly through BCG and MV130 (13). Building on that position, this review takes a translational, evidence-graded perspective and integrates three axes that a viewpoint format could not fully develop. First, we compare inducers and delivery routes (BCG, *β*-glucan, polybacterial immunotherapies, and viral vectors across intranasal, sublingual, and inhaled administration) to show how platform choice governs the quality and location of trained immunity. Second, we grade the clinical evidence by population, spanning children with recurrent wheeze, older adults, patients with chronic obstructive pulmonary disease, and immunocompromised hosts, so that certainty and applicability are made explicit rather than assumed. Third, we apply the protection-vs.-immunopathology balance as a critical lens throughout, and consider the biomarkers and patient-selection strategies needed to steer benefit over harm. Together, these axes frame trained immunity-based mucosal immunotherapy not as a uniform promise but as a set of platform- and population-specific decisions with a defined translational path. This is a narrative, critically appraised review rather than a systematic one. We searched PubMed and Web of Science for English-language literature published between 2011 and 2026, combining terms for trained immunity, innate immune memory, mucosal or airway immunity, and the individual inducers and respiratory pathogens discussed here. Throughout, we prioritized primary randomized and mechanistic studies over secondary sources and applied GRADE-style reasoning to the clinical evidence.

## 2 Mechanistic basis of mucosal and airway trained immunity

### 2.1 Epigenetic and metabolic core

Trained immunity describes a functional state in which innate immune cells, after an initial encounter with a microbial or metabolic stimulus, mount a heightened response to later and often unrelated challenges. This state is written into the cell through coordinated epigenetic and metabolic changes rather than gene rearrangement, and it can be established both centrally, in bone marrow progenitors, and peripherally, in mature tissue cells such as alveolar macrophages (Figure 1) (5). Understanding where and how these programs are laid down is a prerequisite for judging which respiratory platforms will work, in whom, and for how long; the mechanistic features summarized here therefore recur throughout the platform and clinical sections that follow.

**Figure 1:**
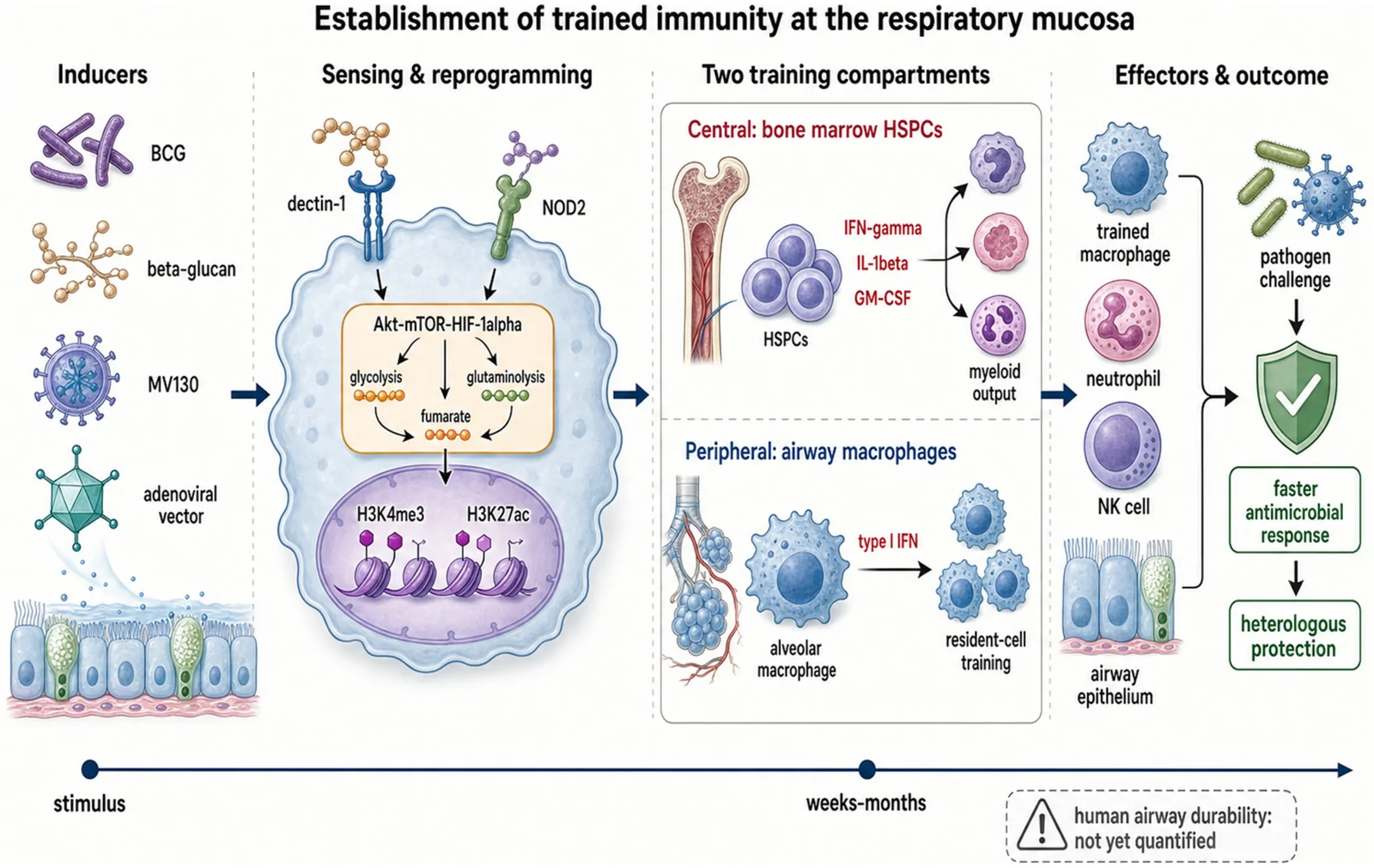
Establishment of trained immunity at the respiratory mucosa. Inducers (BCG, *β*-glucan, polybacterial MV130, adenoviral vectors) are sensed through pattern-recognition receptors (dectin-1, NOD2), triggering Akt-mTOR-HIF-1*α* glycolysis and glutaminolysis-fumarate signaling that deposit activating chromatin marks (H3K4me3, H3K27ac). Reprogramming occurs centrally in bone marrow hematopoietic stem and progenitor cells (IFN-*γ*-, IL-1β-, GM-CSF-driven myeloid output) and peripherally in airway-resident alveolar macrophages (type I interferon-dependent), yielding trained macrophages, neutrophils, natural killer cells, and epithelial responses that confer heterologous protection. Human durability remains to be quantified.

The core of trained immunity is a durable epigenetic and metabolic rewiring of myeloid cells. The concept was established when brief exposure of monocytes to Candida albicans or its cell-wall *β*-glucan was shown to protect lymphocyte-deficient mice against reinfection through functional reprogramming rather than adaptive memory (14), and when BCG vaccination of healthy volunteers was found to raise monocyte-derived cytokine responses to unrelated pathogens for months afterward (15). Both observations converged on a chromatin mechanism: trained cells accumulate lasting marks of permissive chromatin, notably trimethylation of histone H3 at lysine 4 (H3K4me3) and acetylation of H3K27 at the promoters and enhancers of inflammatory genes, so that a second stimulus is transcribed faster and more strongly (3, 16). These chromatin changes do not stand alone. They are sustained by a coordinated shift in cellular metabolism, in which activation of the Akt-mTOR-HIF-1*α* axis drives a switch toward aerobic glycolysis that supplies both the energy and the biosynthetic intermediates for reprogramming (4). Metabolism feeds back onto the epigenome directly: glutaminolysis replenishes the tricarboxylic acid cycle and raises fumarate, which inhibits the KDM5 histone demethylases and thereby preserves activating H3K4me3 marks, while cholesterol and mevalonate pathway activity provides a parallel route to the trained state (17). A systematic transcriptomic and epigenomic comparison of naïve, tolerized, and trained macrophages defined the distinct enhancer landscapes that separate these states, giving the field a molecular reference for what a trained cell actually is and distinguishing trained immunity from endotoxin tolerance (18).

### 2.2 Central vs. peripheral trained immunity

Trained immunity operates at two anatomically distinct levels, a distinction that matters greatly for respiratory prophylaxis. Because mature myeloid cells are short-lived, sustained protection requires reprogramming of hematopoietic stem and progenitor cells (HSPCs) in the bone marrow, a phenomenon termed central trained immunity. Administration of *β*-glucan expands myeloid progenitors and biases hematopoiesis toward granulomonocytic output through interleukin-1β and granulocyte-macrophage colony-stimulating factor signaling, with accompanying shifts in glucose and cholesterol metabolism (19), and BCG that reaches the marrow reprograms HSPCs in an interferon-*γ*-dependent manner to generate epigenetically modified macrophages with enhanced antimicrobial capacity, an effect transferable by bone marrow transplantation and therefore genuinely durable (20). In one murine study, alveolar macrophages acquired a trained phenotype that depended on metabolic rewiring and type I interferon signalling (6). This type I interferon requirement was shown in that specific model, and it should not be generalized to trained responses induced by other microbial insults, in which the signalling requirements differ. The origin of these trained airway cells varies and carries functional consequences: alveolar macrophages trained by adenoviral vectors or by MV130 are largely of tissue-resident or embryonic origin, whereas those arising after influenza infection are recruited from bone marrow monocytes, and lung tissue-resident training induced by microbial stimuli such as lipopolysaccharide can confer differential protection against pneumococci and SARS-CoV-2 (21, 22).

### 2.3 The effector repertoire and its innate–adaptive interface

The effector repertoire of trained immunity extends well beyond the macrophage. BCG vaccination in humans induces long-lasting functional reprogramming of neutrophils, with heightened activation markers (CD11b, CD66b), enhanced degranulation, phagocytosis, and reactive-oxygen production that persist for at least three months and track with genome-wide H3K4me3 changes (23). Natural killer cells contribute a further layer, providing non-specific protection through enhanced cytokine output for up to three months after BCG exposure (24). Structural and stromal cells, including airway epithelium, are increasingly recognized as carriers of innate memory, broadening the cellular basis of local protection at the mucosa. Importantly, trained airway macrophages do not act in isolation from adaptive immunity. After respiratory viral infection, the priming of long-lived, defence-ready alveolar macrophages requires help from local effector CD8⁺ T cells acting through interferon-*γ*, and the trained macrophage phenotype is then maintained within a tissue niche shaped by adaptive signals (25). This defines a bidirectional innate–adaptive feedback loop rather than two parallel systems: tissue-resident memory lymphocytes license and sustain innate memory, while trained macrophages supply the local cytokine and metabolic context that supports resident lymphocyte persistence. Mucosal immunotherapies therefore depend on this innate–adaptive crosstalk to consolidate durable epigenetic memory at the airway mucosa, and they are not purely antigen-agnostic, standalone tools. This cellular diversity means that the net effect of any given inducer reflects a composite of trained macrophages, granulocytes, lymphoid innate cells, and epithelial responses, whose balance is shaped by the stimulus and its route of delivery, and whose regulatory as well as inflammatory components determine whether the outcome favors the host.

The input that forms the innate memory of the airway is not limited to the respiratory tract. Central training of bone-marrow progenitors is influenced by a distal gut–lung axis, in which microbiota-derived short-chain fatty acids reprogram myelopoiesis and set the functional tone of monocyte-derived airway macrophages. Dietary fermentable fibre, acting through this axis, reshapes patrolling-monocyte output and limits neutrophil-driven immunopathology during influenza (26). Diet and the intestinal microbiome are therefore modifiable determinants of the trained state on which a mucosal immunotherapy builds. Additionally, preliminary evidence suggests that trained hematopoietic progenitors may extend to the megakaryocytic lineage, potentially yielding “trained platelets” with altered inflammatory profiles, though their direct role in respiratory mucosal memory requires further clinical validation (27).

Several determinants set the quality, location, and durability of the trained response, and these determinants form the mechanistic bridge to platform design. The nature of the stimulus dictates the receptor engaged and the downstream program, with *β*-glucan acting through dectin-1 and the noncanonical Raf-1 pathway and BCG through NOD2-dependent sensing that deposits H3K4me3 at cytokine and Toll-like receptor promoters (14, 15). The route of administration governs whether central or peripheral training predominates and whether innate memory is deposited in the airway or only in the circulation. Dose, timing, and host factors such as age, metabolic state, and prior infection history further modulate the amplitude and persistence of the effect, and the same machinery can be driven toward either enhanced responsiveness or tolerance depending on context. From a translational standpoint, these variables constitute the levers available to a vaccine designer: matching an inducer and a delivery route to the desired anatomical compartment is the central engineering problem for trained immunity-based mucosal immunotherapy. Key gaps remain in defining how durably human airway training persists, how to measure it non-invasively, and how central and peripheral programs interact after mucosal delivery, and these questions frame the platform-specific evidence examined next.

## 3 Platforms and delivery routes: a comparative appraisal

The inducers that generate trained immunity are chemically and biologically diverse, and each engages the innate immune system through a characteristic receptor, cellular target, and anatomical footprint. For respiratory prophylaxis, the practical question is not simply whether an agent trains innate cells but where it deposits innate memory and how long that memory lasts. The four platform families with the strongest evidence [live mycobacteria, fungal *β*-glucan and other pattern-recognition-receptor (PRR) ligands, inactivated polybacterial preparations, and viral vectors] differ markedly on these axes, and the route of delivery often matters as much as the agent itself. Comparing them on mechanism, delivery route, durability, and translational maturity (Figure 2; Table 1) clarifies which combinations are ready for clinical development and which remain preclinical.

**Figure 2:**
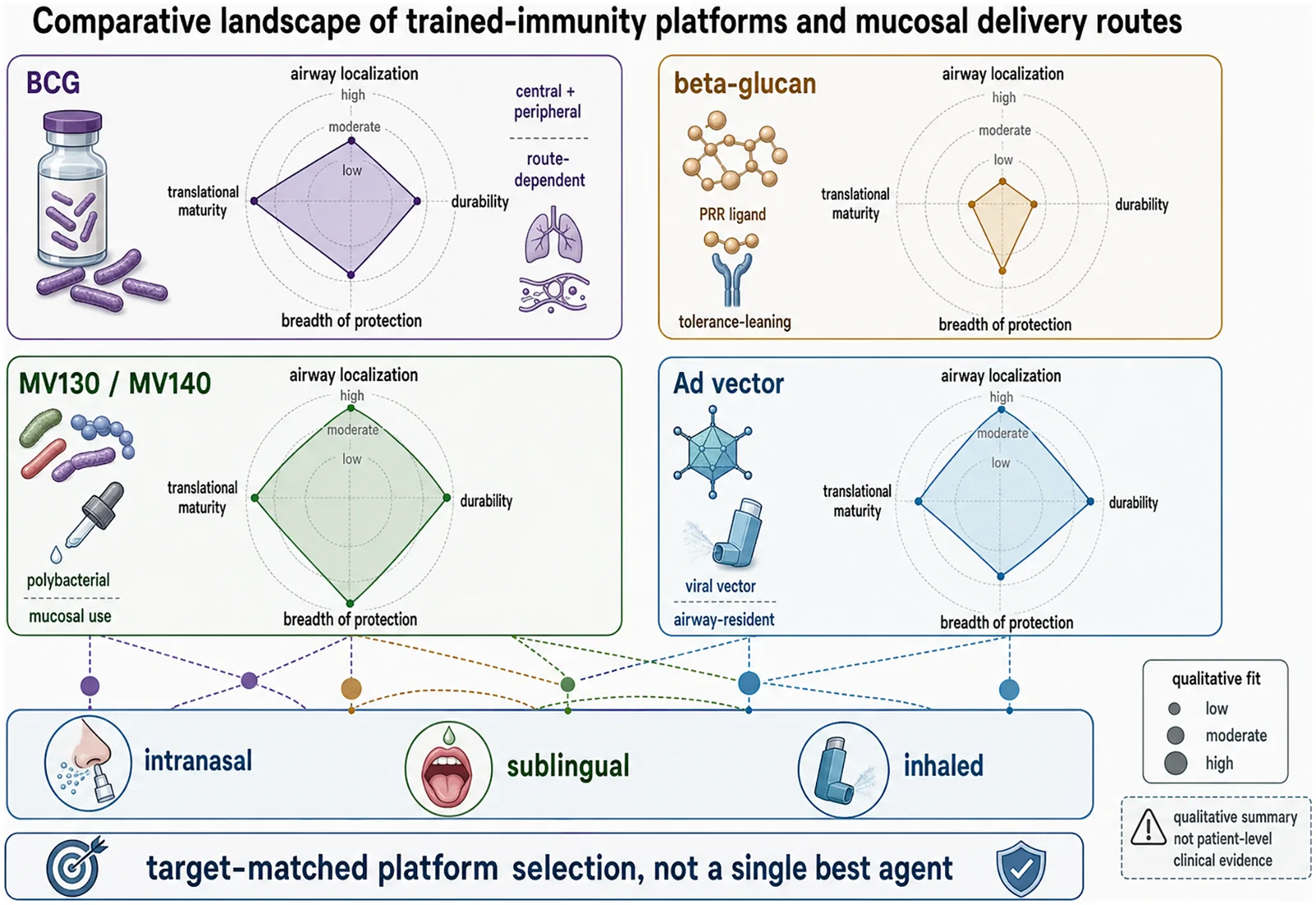
Comparative landscape of trained-immunity platforms and mucosal delivery routes. Radar panels rate BCG, *β*-glucan, MV130, and adenoviral vectors on airway localization, durability, breadth of protection, and translational maturity; a delivery-route band (intranasal, sublingual, inhaled) maps route suitability to each platform. The figure emphasizes target-matched platform selection rather than a single optimal agent.

**Table 1:** Platforms and delivery routes for trained immunity-based mucosal immunotherapy.

Platform (inducer)	Representative agent	Delivery route(s)	Principal mechanism	Training compartment	Durability	Translational maturity	Key evidence
Live mycobacteria	BCG	Intradermal/intravenous/ aerosol-mucosal	Monocyte + HSPC reprogramming (NOD2, H3K4me3); IFN-*γ*–dependent central training	Central + peripheral (route-dependent)	Months to >1 year	High (licensed; routes under study)	(10, 28, 49)
PRR ligand	*β*-glucan	Oral/systemic (airway experimental)	Dectin-1 → Akt-mTOR-HIF-1*α* glycolysis; disease tolerance via regulatory neutrophils	Central + peripheral	Weeks to ∼1 month (model-dependent)	Low–moderate (supplement trials)	(11, 32)
Inactivated polybacterial	MV130/MV140	Sublingual/intranasal	Lymphocyte-independent trained immunity; metformin-sensitive metabolic shift	Central + airway	Sustained during dosing	Moderate–high (phase 3; in clinical use)	(9, 33, 52)
Viral vector	Adenoviral (Ad5, ChAd)	Inhaled/intranasal/ oropharyngeal	Antigen priming + alveolar macrophage training; metabolic/epigenetic rewiring	Peripheral (airway-resident)	Durable local (months)	Moderate (trials ongoing)	(8, 37, 38)

Evidence is graded by study type and annotated by pathogen/trigger and CAP relevance, to distinguish direct bacterial-CAP evidence from indirect (viral or endotoxin) support.

BCG is the prototypical inducer, and its behavior illustrates how delivery route reshapes protection. Parenteral BCG reprograms circulating monocytes and, when it reaches the bone marrow, educates hematopoietic stem cells to produce trained macrophages with enhanced antimicrobial function. Route determines the anatomical reach of this effect: in rhesus macaques, intravenous rather than intradermal BCG generated markedly stronger airway and tissue T cell responses and near-complete protection, with 9 of 10 animals highly protected and 6 showing no detectable infection after virulent tuberculosis challenge (28), and comparable protection was achieved even in simian immunodeficiency virus-infected animals (29). Beyond mycobacterial disease, subcutaneous BCG enhanced innate control of pulmonary Streptococcus pneumoniae through locally trained, neutrophil-dependent responses (30). Aerosol or mucosal delivery is of particular interest for airway protection, because it targets lung-resident cells directly rather than relying on spillover from systemic training, although optimal dosing and the safety of mucosal live BCG in humans remain under study. The heterologous reach of BCG documented across preclinical and human studies spans several respiratory viruses, including respiratory syncytial virus and influenza A virus, which supports its relevance to the viral etiologies that dominate childhood wheeze and seasonal respiratory illness, even as protection remains pathogen- and context-dependent (12).

*β*-Glucan and related PRR ligands offer a defined, non-living alternative that trains myeloid cells through dectin-1 and downstream metabolic reprogramming. *β*-Glucan expands and reprograms bone marrow progenitors (19) and confers protection against experimental tuberculosis through an interleukin-1-dependent program (31), establishing proof of concept for a synthetic inducer. Its respiratory relevance was sharpened by evidence that *β*-glucan protects against influenza A virus principally by promoting disease tolerance (reprogramming granulopoiesis to generate regulatory neutrophils that limit immunopathology) rather than by accelerating viral clearance (11). This mechanistic signature distinguishes *β*-glucan from inducers that act mainly on resistance, and it carries direct implications for which clinical outcomes such an agent is likely to improve. Human supplementation trials point in a consistent, if modest, direction: in community-dwelling older adults, daily yeast *β*-1,3/1,6-glucan reduced the number of confirmed upper respiratory tract infections relative to placebo and tended to shorten symptom duration (32), and controlled studies in healthy adults reported reduced severity of early upper respiratory symptoms. These trials use oral rather than airway delivery and industry-supplied preparations, so their effect sizes should be read cautiously, but they establish that a chemically defined trained-immunity inducer can influence clinically relevant respiratory endpoints in humans.

Inactivated polybacterial mucosal immunotherapies represent the platform closest to routine clinical use. MV130, a sublingual whole-cell preparation dominated by Gram-positive with a minority of Gram-negative inactivated bacteria (the sublingual route is used in human clinical trials, whereas the intranasal route is confined to pre-clinical rodent models). It protects against experimental viral respiratory infection through a trained-immunity mechanism that is lymphocyte-independent and can be abrogated by pharmacological inhibition of innate metabolic reprogramming with metformin (7). The same trained-immunity-based vaccine concept has been validated at a different mucosal site, where the related sublingual preparation MV140 achieved freedom from recurrent urinary tract infections in 56%–58% of treated women vs. 25% on placebo over nine months in a randomized trial (33), and combinations with fungal components have been proposed to broaden coverage (34). Because these products are inactivated, delivered mucosally, carry an established safety record, and are already in clinical use for recurrent infections, they provide the most direct template for trained immunity-based respiratory prophylaxis.

Viral-vectored and next-generation platforms combine antigen-specific immunity with potent innate training and are well suited to mucosal delivery. Inhaled adenoviral-vectored vaccines reprogram human alveolar macrophages in a manner that intramuscular delivery does not, coupling classical adaptive priming to durable local innate memory (8), and a chimpanzee adenoviral vaccine engineered to train alveolar macrophages improved cell-intrinsic control of mycobacteria (35). The durability of airway-macrophage training is notable: influenza-trained, mucosal-resident alveolar macrophages confer long-lasting, tissue-specific functional changes in the lung (36), illustrating both the promise and, as discussed later, the potential risks of persistent local reprogramming. Clinical translation is advancing on multiple fronts: an inhaled aerosolized adenoviral SARS-CoV-2 vaccine used as a heterologous booster elicited mucosal and systemic responses with a dose-sparing, well-tolerated profile at one-fifth the intramuscular dose (37), an intranasal adenoviral vaccine induced mucosal IgA-secreting responses and met a superiority criterion for neutralizing titers in a phase 3 comparison (38), and oropharyngeal adenoviral spray immunization protected against heterologous SARS-CoV-2 (39). Live attenuated mucosal vaccines against strictly respiratory pathogens, exemplified by the intranasal pertussis vaccine BPZE1, further show that the airway route can generate the broad secretory IgA responses that injectable formulations fail to induce, with more than 90% of recipients seroconverting for nasal IgA against at least one pertussis antigen (40). Mucosal IgA, neutralizing titres, and nasal seroconversion are classical measures of adaptive immunity. They remain mechanistically relevant here because the same platforms also engage pattern-recognition receptors that remodel the local innate microenvironment: inhaled adenoviral vectors durably reprogram airway-resident alveolar macrophages (6, 8). Their clinical success therefore reflects a dual action, in which adaptive priming and innate training are induced together rather than adaptive immunity alone.

The respiratory relevance of trained immunity has now been tested against several pathogens that dominate lower-airway disease. For influenza, a prior infection recruits monocyte-derived alveolar macrophages that persist in the lung and confer prolonged heterologous protection against subsequent *Streptococcus pneumoniae* infection (21). Against S. pneumoniae itself, inhaled lipopolysaccharide induces lung-resident alveolar-macrophage training that enhances bacterial clearance through metabolic rewiring and type I interferon signalling (6). Among multidrug-resistant Gram-negative pathogens, a single intranasal immunization with inactivated whole-cell *Acinetobacter baumannii* trains alveolar macrophages and confers rapid heterologous protection that extends to *Pseudomonas aeruginosa* and *Klebsiella pneumoniae* (41).Specifying where training does and does not protect across these pathogens, rather than treating “respiratory infection” as a single entity, is essential to the evidence-graded reading advanced here.

Set side by side, these platforms trade off along consistent lines. Live mycobacteria and viral vectors deliver strong, durable training but raise manufacturing, genetic-stability, and safety considerations that are heightened for mucosal live delivery; inactivated polybacterial preparations offer favorable safety and an established clinical footprint at the cost of less well-defined potency; and purified ligands such as *β*-glucan provide chemical definition and scalability while acting predominantly on tolerance rather than resistance (42). Delivery route cuts across all four families as an independent determinant, with intranasal, sublingual, and inhaled administration each favoring different compartments and effector profiles. Platform choice should therefore be reasoned backward from the clinical goal: the pathogen spectrum to be covered, the compartment where protection is needed, and the acceptable safety margin for the target population. The key gaps are the absence of head-to-head comparisons across platforms and routes, the lack of standardized potency assays for trained immunity, and limited human data on the durability of airway training, all of which must be resolved before rational platform selection can guide the population-specific clinical decisions examined next.

Mucosal induction of trained immunity also has a macro-structural dimension that purely cellular accounts overlook. Beyond reprogramming individual macrophages and granulocytes, airway delivery of microbial inducers can drive stromal activation and leukocyte recruitment that nucleate inducible bronchus-associated lymphoid tissue (iBALT). These ectopic follicular structures organize local T- and B-cell responses and can sustain protective respiratory immunity even without conventional secondary lymphoid organs (43). Mucosal trained-immunity inducers may therefore do more than stimulate cells transiently: by nucleating iBALT, they help build the regional niches that store long-term airway memory, giving the innate reprogramming emphasized throughout this review a lasting structural basis.

## 4 Clinical evidence by population: an evidence-graded synthesis

The clinical case for trained immunity-based mucosal immunotherapy is strongest when read population by population rather than as a single verdict, because certainty of evidence, effect size, and applicability differ sharply across patient groups. Randomized trials now exist for children with recurrent wheeze and for older adults, observational and phase 3 data support use in chronic airway disease, and early studies address immunocompromised hosts, but the maturity of this evidence is uneven. Grading it explicitly (Table 2), rather than pooling heterogeneous endpoints, is the analytical step that distinguishes a translational appraisal from advocacy, and it directly addresses a gap left by opinion-level treatments of the field.

**Table 2:** GRADE-style summary of clinical evidence by population.

Population	Intervention (route)	Best available evidence	Key reported effect	Certainty (GRADE)	Main reason for downgrading
Children with recurrent wheeze	MV130 (sublingual)	Phase 3 RCT, *n* = 120 (9); real-world (44)	↓ number and duration of wheezing attacks	Moderate–High	Single trial; durability unknown
Older adults	BCG (intradermal)	RCT ACTIVATE (10); BRACE (49); meta-analysis (50)	New infections 42.3% → 25.0% (ACTIVATE); COVID-19 benefit not reproduced	Moderate	Inconsistency across trials; context-dependent
COPD	MV130 (sublingual)	Phase 3 RCT, *n* = 198 (52)	↓ ∼30% exacerbations; shorter duration; ↓ antibiotics	Moderate	Conference-abstract stage; awaiting full publication
Immunocompromised (B-cell malignancy)	MV130 (sublingual)	Single-center pilot, *n* = 15 (53)	Clinical improvement; ↓ antibiotics; specific IgA/IgG	Low	Small, uncontrolled; hypothesis-generating

### 4.1 Children with recurrent wheeze

*Evidence Certainty Profile: Moderate–High. Single phase 3 RCT (n* *=* *120) plus real-world data; durability after cessation unknown.*

Children with recurrent wheeze and recurrent respiratory tract infections have the most direct randomized evidence. In a phase 3, double-blind, placebo-controlled trial enrolling 120 children under three years with three or more wheezing attacks in the previous year, sublingual MV130 given daily for six months reduced the number and duration of wheezing attacks over the following year relative to placebo (9). The clinical logic is compelling because most early-childhood wheezing is triggered by viral infection, chiefly rhinovirus and respiratory syncytial virus, for which few preventive options exist, and because a mucosal, antigen-agnostic approach sidesteps the antigenic diversity of the responsible viruses. The mechanistic underpinning is consistent with the trial outcome, since intranasal MV130 (pre-clinical, intranasal) confers lymphocyte-independent, trained-immunity-dependent protection against experimental viral respiratory infection in mice (7). Real-world data in children and adults are consistent with the randomized result, reporting fewer infections and reduced antibiotic use after MV130 (44). Certainty here is moderate to high for the wheezing endpoint, although the durability of protection after treatment stops, the optimal dosing schedule, and generalizability beyond the trial population remain to be defined.

### 4.2 Older adults

*Evidence Certainty Profile: Moderate. ACTIVATE positive; BRACE and meta-analysis inconsistent; effect context-dependent.*

Older adults represent the population in which non-specific protection was first tested in a dedicated trial. The ACTIVATE study, a phase 3 double-blind trial in elderly patients discharged from hospital, showed that a single BCG vaccination lengthened the time to first infection and lowered the incidence of new infections from 42.3% after placebo to 25.0% after BCG, with most protected events being respiratory infections of probable viral origin (10). This built on earlier controlled human data that BCG protects against experimental viral infection through trained-immunity cytokine programs (15). The clinical foundations of this field in fact predate the modern trained-immunity concept. The first randomized controlled trial of BCG given at birth to low-birth-weight infants in Guinea-Bissau reported that neonatal-period mortality fell markedly, driven chiefly by fewer cases of neonatal sepsis and respiratory infection, providing the first controlled evidence that BCG exerts heterologous, non-specific protection against unrelated infectious death (45); a later randomized trial in the same setting confirmed this early-life benefit (46). Together with systematic reviews of the non-specific effects of BCG (47, 48), these early observations established the morbidity- and mortality-level rationale on which the mechanistic and prophylactic work reviewed here was later built. The pandemic prompted larger tests of the same principle, and results have been mixed. The BRACE trial evaluated BCG against febrile and respiratory illness in adults, with primary outcomes that tempered earlier optimism (49), and a meta-analysis of BCG revaccination against COVID-19 pooling thirteen randomized trials and 13,939 participants found no consistent benefit against SARS-CoV-2 (50). Longstanding observational data that neonatal BCG reduces hospitalization for respiratory infection and sepsis, by roughly 40% and 36% respectively in one large analysis of more than 460,000 admissions, frame the biological plausibility (51). Taken together, the heterologous protection seen in older adults is best regarded as of moderate and context-dependent certainty, robust for endpoints resembling the seasonal respiratory infections captured in ACTIVATE but unproven for any single emerging pathogen.

### 4.3 Chronic obstructive pulmonary disease

*Evidence Certainty Profile: Moderate. Phase 3 RCT (n* *=* *198); currently conference-stage, awaiting full peer-reviewed publication.*

Chronic obstructive pulmonary disease (COPD) is a rational target because most exacerbations are triggered by respiratory infection. A phase 3 randomized, double-blind, placebo-controlled trial of sublingual MV130 in 198 patients with moderate-to-severe COPD, who had experienced three exacerbations or two with one hospitalization in the prior year, reported roughly a 30% reduction in total exacerbations over eighteen months, a shorter median duration of exacerbations (10 vs. 20 days), and reduced antibiotic use (12 vs. 29 days), with a favorable safety profile and fewer adverse events than placebo (52). Because these results have so far been reported principally in conference form, the certainty is currently moderate pending full peer-reviewed publication, but the direction and magnitude are consistent with the mechanism and with the pediatric and elderly data.

### 4.4 Immunocompromised and other special populations

*Evidence Certainty Profile: Low. Single-center pilot (n* *=* *15); hypothesis-generating.*

Immunocompromised and other special populations are the least studied yet arguably among the most in need. A single-center pilot study of sublingual MV130 in fifteen patients with B-cell hematological malignancies and recurrent respiratory infections reported clinical improvement in the majority, a fall in antibiotic consumption and infection-related healthcare visits, and detectable MV130-specific IgA and IgG responses despite the underlying antibody defect (53), and mucosal trained immunity-based approaches have been proposed specifically for patients on immunosuppression, in whom antigen-specific vaccination often responds poorly (54). Evidence here is low certainty and hypothesis-generating, but the favorable safety profile and the mechanistic independence from adaptive immunity make further controlled trials attractive.

### 4.5 Why the very young and the old need it most

*Evidence Certainty Profile: mechanistic rationale (not an efficacy tier); a unifying explanation for age-specific need.*

Why the very young and the old benefit most has a common immunological explanation. In older adults, susceptibility to respiratory viruses is worsened by a kinetic delay in innate antiviral signalling. Aged hosts mount lower and later type I interferon responses during the first days of infection, which allows unchecked early viral replication and a damaging late-phase inflammatory surge (55, 56). This “interferon gap” is compounded by thymic involution and the loss of peripheral cellular immunity that accompany immunosenescence, so adaptive reinforcement arrives too slowly to help. In young infants, the adaptive arm is immunologically naïve and structurally prone to aberrant type 2 skewing, again leaving early control to innate defences. Mucosal trained immunity suits both settings because it acts as a cellular adjuvant that primes airway-resident cells to a defence-ready state, shortening the interval to early antiviral effector function and narrowing the interferon gap without relying on a competent adaptive compartment (55). The pediatric wheeze and older-adult signals reviewed above therefore follow from restoring early innate kinetics in hosts whose defect lies precisely there, rather than standing as isolated clinical observations.

Read across populations and graded with a common framework such as GRADE (57), the evidence forms a gradient rather than a uniform endorsement (Table 2). Certainty is highest for MV130 in pediatric recurrent wheeze, moderate for BCG in older adults and for MV130 in COPD, and low but promising in immunocompromised hosts; a systematic review and meta-analysis of mucosal respiratory vaccines more broadly, spanning 65 studies and more than 229,000 participants, supports enhanced local immunogenicity while underscoring heterogeneity in protective endpoints (58). Two cross-cutting lessons emerge. First, effect and certainty travel with the specific agent, route, and endpoint, so claims must be tied to the exact intervention tested rather than to trained immunity in general (12). A related caveat is that much of the polybacterial-immunotherapy evidence derives from a single product family and its associated research network, so independent replication by unaffiliated groups is needed before these results can be generalized. Second, non-specific effects vary with host factors including age and sex, which complicates extrapolation between populations (47), a caution reinforced by systematic reviews that flagged methodological limitations in the older non-specific-effects literature (48). The translational priority is therefore population-specific: adequately powered trials with standardized clinical endpoints in older adults, COPD, and immunocompromised patients, harmonized outcome definitions to allow pooling, and predefined subgroup analyses that respect the host-dependent nature of the effect. These evidentiary needs set up the central safety question examined next, namely how to ensure that enhancing innate responsiveness helps rather than harms.

## 5 The double-edged sword: protection vs. immunopathology

Enhancing innate responsiveness is not intrinsically safe, and the same reprogramming that improves host defense can, under the wrong conditions, injure the host. This tension is central to any clinical use of trained immunity-based mucosal immunotherapy, yet it is often underplayed in advocacy for the approach. Three considerations define the safety landscape: whether protection is achieved by resisting the pathogen or by tolerating it, whether training can become maladaptive and drive inflammation or fibrosis, and how conflicting real-world results should temper expectations. Together these considerations motivate a biomarker-guided strategy for selecting patients in whom benefit is likely to outweigh harm.

The distinction between host resistance and disease tolerance reframes what protection means for a trained-immunity agent. Resistance reduces pathogen burden, whereas tolerance limits the damage a given burden causes without necessarily clearing it faster. *β*-Glucan protects against influenza A virus largely through the tolerance arm, reprogramming granulopoiesis to generate regulatory neutrophils that preserve lung tissue integrity rather than accelerating viral clearance (11). This evidence is derived predominantly from murine models, and its extrapolation to humans remains to be established; the distinction is nonetheless clinically instructive as a hypothesis to test rather than an established human mechanism. Innate memory in alveolar macrophages after prior viral infection can likewise ameliorate a subsequent heterologous infection, illustrating a beneficial legacy, but the same cells sit at the fulcrum between protection and pathology (59). This mechanistic split matters clinically: an intervention that improves tolerance may reduce symptoms and tissue damage even when it does not lower transmission, which changes both the endpoints worth measuring and the populations most likely to benefit.

Trained immunity can also become maladaptive when it is induced by the wrong stimulus or sustained beyond its useful window. The clearest evidence comes from cardiometabolic disease, where a Western diet triggers NLRP3-dependent reprogramming of myeloid progenitors that produces a durable proinflammatory state persisting even after the dietary stimulus is withdrawn (60), and broader analyses place trained immunity among the drivers of chronic inflammatory pathology when its induction is inappropriate, spanning atherosclerosis, autoinflammatory, and neurodegenerative conditions (61). In the lung specifically, excessive or persistent innate activation can shift the balance from protective host defense toward immunopathology, inflammatory tissue damage, and fibrotic remodeling, so that the same durable airway-macrophage reprogramming that underlies protection could, in a susceptible host, sustain injury. A concrete cellular example of this hazard is the neutrophil. Maladaptive training of granulomonocytic progenitors can reprogram granulopoiesis and yield neutrophils with heightened effector output, including a greater capacity for neutrophil extracellular trap formation (62). During secondary bacterial or viral pneumonia, unrestrained NETosis becomes a direct cause of endothelial injury, microvascular thrombosis, and fibrotic remodeling rather than a protective mechanism. The same progenitor-level reprogramming that can strengthen antimicrobial defence may therefore, in a susceptible host, turn trained neutrophils into agents of lung damage. The protection-vs.-immunopathology balance is thus set across the trained myeloid output as a whole, not in the macrophage compartment alone. The therapeutic relevance of this pathway is underscored, with an important directional caveat, by the CANTOS trial. Blocking interleukin-1β reduced recurrent cardiovascular events independently of lipid lowering, but it also increased fatal infections and sepsis (63). CANTOS therefore shows that the IL-1β axis engaged by trained immunity is potent and clinically modifiable. For mucosal immunotherapy, the implication is local, controlled optimization rather than systemic suppression, so that airway defence is enhanced without the systemic immunosuppression that caused the infectious harm seen with sustained IL-1β blockade. In the allergic airway, trained immunity may amplify aberrant type 2 responses and worsen disease, offering a further example of context-dependent harm that is directly relevant to the pediatric wheeze population in which MV130 is used (64).

Conflicting clinical results for BCG against COVID-19 provide a cautionary illustration of context dependence. Early enthusiasm rested on the plausible premise that BCG-induced trained immunity might bridge protection before specific vaccines arrived (12), yet larger randomized trials and meta-analysis failed to reproduce a consistent benefit against SARS-CoV-2 (50). Preclinical data similarly show that BCG-induced training does not protect against every respiratory pathogen, failing for instance against some influenza challenges despite a measurable trained phenotype *ex vivo* (65). The lesson is not that trained immunity is ineffective but that its outcome is contingent on the pathogen, the host, the dose, and the timing, so that protection demonstrated in one setting cannot be assumed in another. Related work on sepsis reinforces the point from the opposite direction: the same reprogramming machinery underlies both trained immunity and the immunoparalysis that follows severe infection, and strategies that push cells toward the trained state are being explored to reverse that suppression (66).

Managing this double-edged behavior in the clinic requires biomarkers and patient selection rather than blanket application. Because trained-immunity programs are heterogeneous at the single-cell level and their signatures appear in inflammatory and infectious disease states alike, molecular readouts could in principle identify who will respond protectively and who risks harm (67). Candidate markers span several layers and could be combined into a selection algorithm (Figure 3): epigenetic signatures such as H3K4me3 and H3K27ac enrichment at inflammatory gene promoters in circulating myeloid cells; metabolic readouts of the glycolytic and glutaminolytic shift, including lactate production and fumarate accumulation; baseline and post-induction inflammatory indices such as interleukin-1β, interleukin-6, tumor necrosis factor, and C-reactive protein; and host descriptors including age, metabolic and inflammatory status, and prior infection history; and, given the mucosal focus of these therapies, localized non-invasive respiratory read-outs such as nasal epithelial swabs, induced-sputum profiles, and fractional exhaled nitric oxide (FeNO), which capture the airway compartment that systemic markers cannot reflect. A pragmatic decision flow would stratify candidates by baseline inflammatory tone, favor agents that act through tolerance in those prone to immunopathology, and monitor a post-induction marker panel to detect maladaptive training early. Practical selection will therefore combine host factors already known to shape non-specific effects with emerging epigenetic and metabolic markers of the trained state, though each candidate marker still requires prospective validation against clinical endpoints. The translational agenda that follows is concrete: safety endpoints, including markers of immunopathology, must be built into prophylaxis trials from the outset; the resistance-vs.-tolerance mechanism of each agent should be defined so that endpoints match the expected mode of action; and predictive biomarkers must be validated prospectively rather than assumed. Three questions remain the pivot on which safe clinical translation turns: how to measure maladaptive training before it causes harm, how to set a safe therapeutic window for each platform, and how host state modifies the benefit-risk balance. They lead directly into the roadmap developed next.

**Figure 3:**
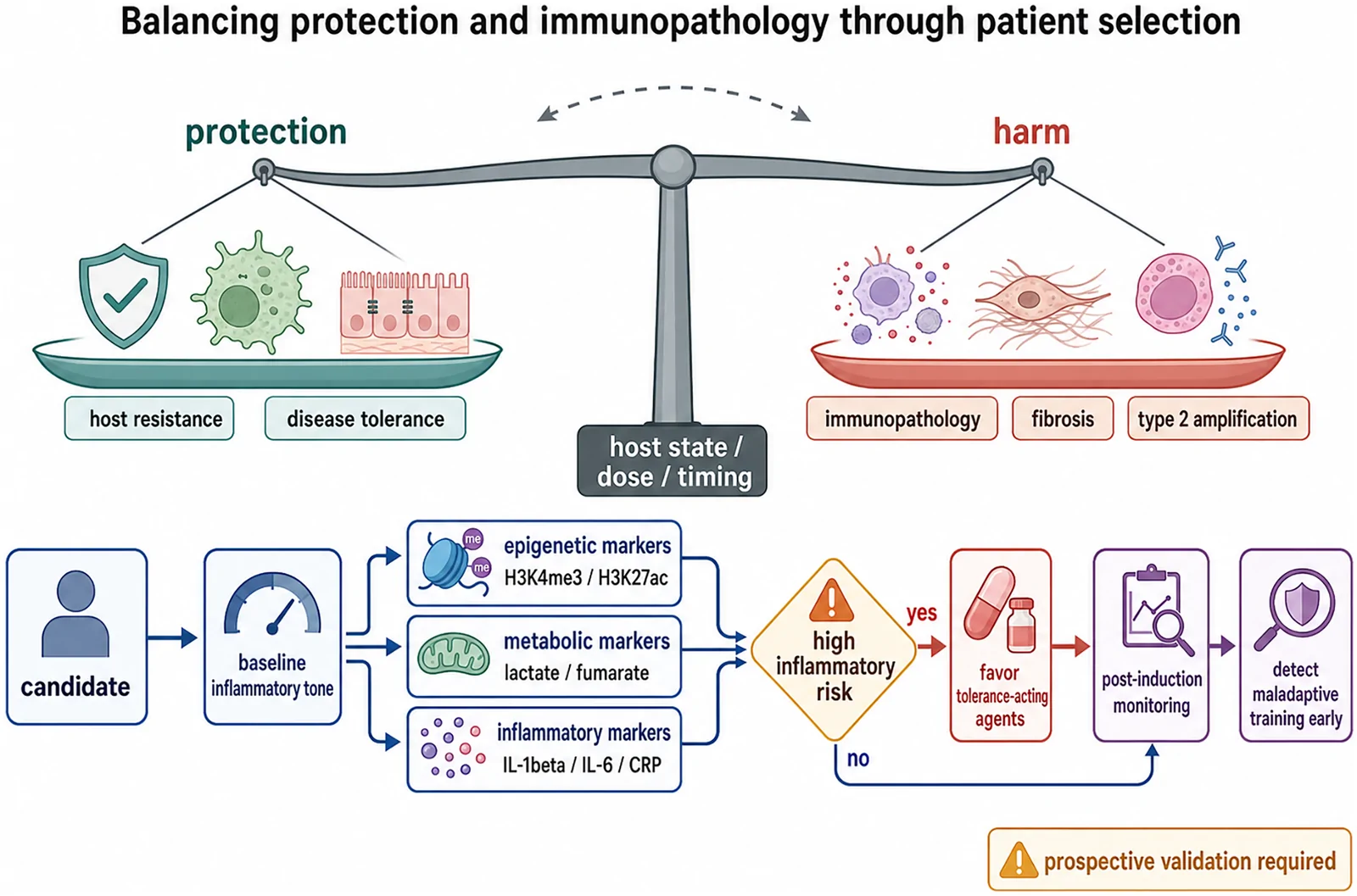
Balancing protection and immunopathology through patient selection. A conceptual balance contrasts protection (host resistance and disease tolerance) with harm (immunopathology, fibrosis, type 2 amplification), pivoting on host state, dose, and timing. A decision flow stratifies candidates by baseline inflammatory tone and epigenetic, metabolic, and inflammatory markers, favors tolerance-acting agents in immunopathology-prone hosts, and monitors a post-induction marker panel; all candidate markers require prospective validation.

## 6 Translational roadmap and open questions

Moving trained immunity-based mucosal immunotherapy from proof of concept to prescription requires solving problems that are practical as much as biological. The clinical signals reviewed above are real but scattered across agents, routes, and populations, and they rest on endpoints that were rarely designed to capture innate-memory effects. A coherent path forward rests on four pillars: standardizing the interventions themselves, designing prophylaxis trials with fit-for-purpose endpoints, addressing regulatory and manufacturing realities, and prioritizing the knowledge gaps whose resolution would most change practice.

Standardization is the foundational requirement. Trained immunity is induced by agents as different as live mycobacteria, purified *β*-glucan, inactivated polybacterial mixtures, and viral vectors, and each is characterized today by inconsistent dosing, schedules, and formulations. Without agreed definitions of what constitutes an adequate trained response, comparisons across products and trials remain unreliable. The field needs validated potency assays that capture the epigenetic and metabolic hallmarks of training, together with reference standards that allow a given lot to be qualified before clinical use (68). Durability is part of this problem: some human training signatures are short-lived, as shown by the transient innate reprogramming after repeated mRNA vaccination (69), which means that schedule and boosting intervals must be defined empirically rather than assumed.

Trial design and endpoint selection are the second pillar, and they must be tailored to the mode of action. Because some agents act through disease tolerance rather than resistance, trials that measure only pathogen load or transmission will miss genuine clinical benefit, whereas trials measuring symptom burden, exacerbations, and tissue-protective outcomes may capture it. Endpoints should be harmonized across studies to permit pooling, graded transparently for certainty, and paired with prespecified safety measures that include markers of immunopathology (57). The practical difficulty of assessing mucosal immune responses in humans, including sampling the airway compartment and interpreting local correlates, is itself a barrier that trial protocols must confront directly (70). Prophylaxis trials in older adults, COPD, and immunocompromised patients should be powered for clinically meaningful infection endpoints and structured to detect host-dependent variation in effect.

Regulatory and manufacturing considerations form the third pillar and shape which platforms can realistically scale. Live and vectored products raise questions of genetic stability, batch consistency, and the safety of mucosal administration, whereas inactivated polybacterial preparations and purified ligands present more tractable manufacturing but less well-defined mechanisms of potency (42). Regulators will require a clear articulation of mechanism, a validated measure of the intended biological effect, and a benefit-risk framework appropriate to prophylactic use in often healthy or vulnerable populations. The argument for treating trained immunity-based products as a distinct vaccine class is useful here, because it invites purpose-built evaluation pathways rather than forcing these agents into frameworks designed for antigen-specific vaccines. In practice, these assets sit ambiguously between established regulatory definitions, straddling live biotherapeutic products, conventional vaccine adjuvants, and immunomodulators. This is precisely why a purpose-built evaluation pathway is needed, rather than forcing them into a single existing category (13).

The priority knowledge gaps follow a logical order from mechanism to clinic (Figure 4). First, the durability and anatomical localization of human airway trained immunity remain poorly quantified, and non-invasive assays to track them are lacking. Second, the determinants that tip training toward protection vs. immunopathology are incompletely mapped, leaving patient selection without validated predictive markers. Third, head-to-head comparisons of platforms and delivery routes are essentially absent, so rational matching of agent to clinical goal rests on inference rather than direct evidence. Fourth, the interaction between centrally and peripherally induced training after mucosal delivery, and its consequences for both efficacy and safety, is not understood. Addressing these questions in sequence would convert a promising but heterogeneous body of work into an evidence base capable of guiding practice. The overarching message is that trained immunity-based mucosal immunotherapy should advance not as a single product or claim but as a disciplined program in which standardized interventions, mechanism-matched endpoints, and prospective biomarkers are developed together.

**Figure 4:**
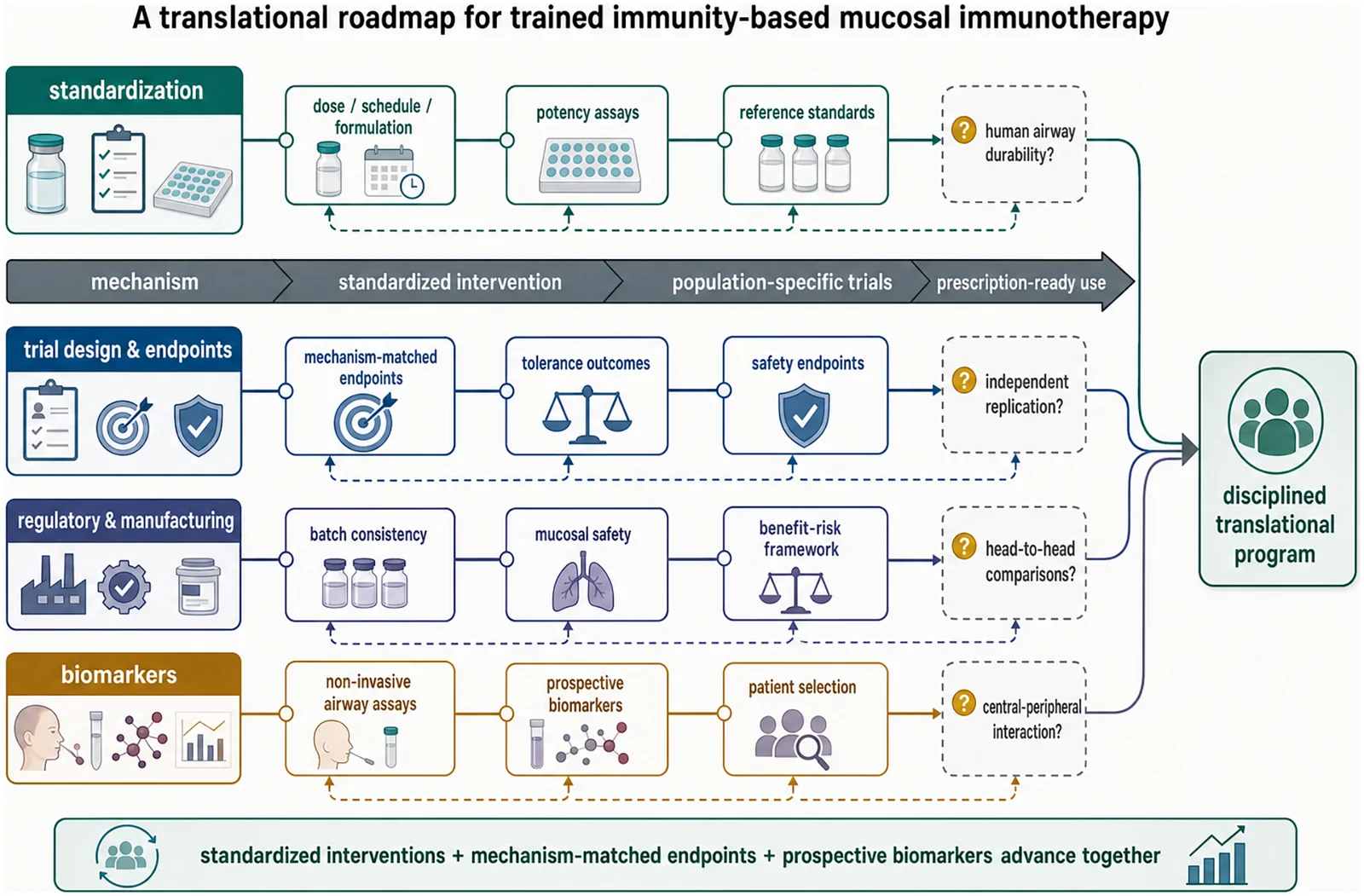
A translational roadmap for trained immunity-based mucosal immunotherapy. Four lanes (standardization, trial design and endpoints, regulatory and manufacturing, biomarkers) are arranged along a timeline with milestones and open questions (e.g., potency assays, tolerance-matched endpoints, independent replication, non-invasive airway-training assays), underscoring that standardized interventions, mechanism-matched endpoints, and prospective biomarkers must advance together.

## 7 Conclusion

Trained immunity-based mucosal immunotherapy is best understood not as a single promise but as a framework of platform- and population-specific decisions. Three factors jointly determine its clinical value: the anatomical site where innate memory is deposited, the mode of protection an agent confers, and the balance between host defense and immunopathology. This framework has practical utility: it explains why delivery route and inducer shape outcomes, it structures the design of prophylaxis trials around mechanism-matched endpoints, it offers a rationale for predicting who will benefit, and it points toward optimizing therapy by matching a platform to a defined clinical goal rather than deploying trained immunity indiscriminately. Realizing this value requires overcoming interlocking challenges. Experimental models must better represent human airway biology and the durability of local training; data from heterogeneous trials and mechanistic studies need integration under harmonized definitions and evidence grading; new methods for non-invasive measurement of airway trained immunity must be adopted; and computational approaches, including machine learning applied to multi-omic signatures, could help resolve the heterogeneity of trained-immunity programs and identify predictive biomarkers of benefit vs. harm. Progress on these fronts feeds back into the framework itself. Standardized models and assays would sharpen the resistance-vs.-tolerance distinction and quantify durability; integrated data and biomarker-guided selection would convert population-specific signals into actionable clinical decisions; and each successful translation would in turn test, refine, and validate the conceptual framework, closing the loop between mechanism and medicine. Positioned this way, trained immunity-based mucosal immunotherapy offers a credible complement to antigen-specific vaccines against respiratory infections, provided its development proceeds as a disciplined program that advances standardized interventions, mechanism-matched endpoints, and prospective biomarkers together.
